# Grossesse hétérotopique à terme: à propos d'un cas

**DOI:** 10.11604/pamj.2014.19.134.5443

**Published:** 2014-10-07

**Authors:** Moncef Chagou, Mohammed Ali Benyahia

**Affiliations:** 1 Service de Gynécologie Obstétrique Cancérologie et Grossesse à haut risque, Maternité Souissi, Université Mohammed V, Rabat, Maroc

**Keywords:** Grossesse extra-utérine, grossesse intra-utérine-avortement, tubo-ovarien, extrauterine pregnancy, intrauterine pregnancy, ovarian tube

## Image en medicine

Nous rapportons le cas d'une patiente âgée de 25 ans, primigeste primipare, sous contraceptif oral depuis 5 ans arrêté 3 mois avant la grossesse. La patiente n'a jamais été suivie pour sa grossesse. A l'admission de la patiente, l'examen a objectivé une hauteur utérine à 30 cm, bruits cardio-foetaux bien perçus. Au toucher vaginal, le col parait effacé à 90% et dilaté à 3 cm. La présentation est céphalique Nous avons réalisé une échographie qui a montré que les biométries correspondaient bien au terme. Devant les décélérations tardives et répétitives du rythme cardio-foetal, une césarienne a été indiquée pour souffrance foetale au début du travail ce qui est a permis l'extraction d'un nouveau né de sexe féminin avec un apgar 10/10. A l'exploration systématique des annexes, nous avons détecté une grossesse extra utérine infundibulaire. Nous avons réalisé un avortement tubo-ovarien, l'anatomopathologie a confirmé la grossesse extra-utérine. Nous avons conclu à une grossesse hétérotopique menée à terme.

**Figure 1:**
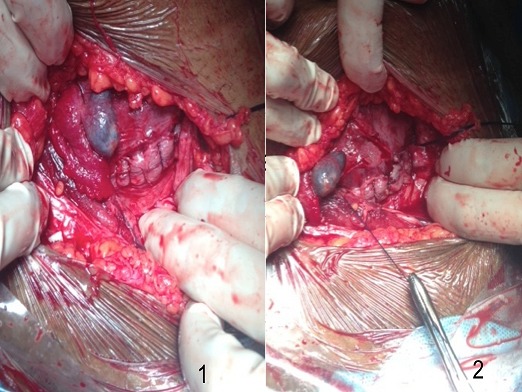
Images per opératoires montrant la grossesse extra-utérine infundibulaire collectée non rompue

